# AMPK activation improves depression-like symptoms in olfactory bulbectomized mice by regulating microglia M1/M2 polarization in the hippocampus

**DOI:** 10.1016/j.bbih.2025.101008

**Published:** 2025-05-11

**Authors:** Takayo Odaira-Satoh, Osamu Nakagawasai, Kohei Takahashi, Masae Shimada, Wataru Nemoto, Koichi Tan-No

**Affiliations:** a Division of Pharmacology, Faculty of Pharmaceutical Sciences, Tohoku Medical and Pharmaceutical University, 4-4-1 Komatsushima, Aoba-ku, Sendai 981-8558, Japan; b Department of Pharmacology, School of Pharmacy, International University of Health and Welfare, 2600-1 Kitakanemaru, Ohtawara, Tochigi 324-8501, Japan

**Keywords:** AICAR, AMPK, Antidepressant, Microglial polarization, Neuronal cell survival, Olfactory bulbectomy

## Abstract

Several studies have reported that the activation of adenosine monophosphate-activated protein kinase (AMPK) in the central nervous system is involved in antidepressant-like effects. We recently demonstrated that AMPK activators like 5-aminoimidazole-4-carboxamide-1-β-d-ribonucleotide (AICAR) and liver hydrolysate containing an AMPK active ingredient can prevent depression-like behaviors in animal models of depression through enhanced cell proliferation in the hippocampal dentate gyrus (DG). However, it remains unclear whether microglia are involved in the antidepressant effects of AICAR in olfactory bulbectomized (OBX) mice, which is a useful animal model of depression. Therefore, in this study, we aimed to determine the mechanism of action of AICAR in OBX mice through various behavioral tests and immunohistochemical test. OBX mice exhibited depression-like behaviors in the tail suspension test (TST), forced swimming test (FST), sucrose splash test (SST), and sucrose preference test (SPT). Immunohistochemical studies revealed decreased hippocampal neuronal cell survival and an imbalance in microglial M1/M2 polarization: increased M1-like phenotype and decreased M2-like phenotype. However, AICAR treatment for 3 weeks improved the OBX-induced prolonged immobility in the TST and FST and decreased grooming time and sucrose intake rate in the SST and SPT, respectively. Chronic AICAR administration also ameliorated the reduction in hippocampal neuronal cell survival and the imbalance in microglia polarization. Our results indicate that activated AMPK improves depression-like behavior by neuroprotection via the regulation of microglial polarity. Thus, AMPK activation offers potential therapeutic avenues for developing novel treatment strategies for neuropsychiatric disorders such as depression.

## Highlights

• AICAR administration improved the OBX-induced depression-like behaviors.
• AICAR prevented the reduction of neuronal cell survival in the hippocampus of DG in OBX mice.
• AICAR prevented the polarization to M1-type microglia in the hippocampus of OBX mice.

## 1 Introduction

Major depressive disorder (MDD) continues to be a serious public health burden, which is persistently undertreated (Smith, 2014). Available evidence indicates that depression is closely associated with chronic neuroinflammatory disorders (You et al., 2011) and changes in inflammation and microglial activation (Yirmiya et al., 2015) and can lead to increased levels of inflammatory cytokines (Milior et al., 2016). An imbalance in the inflammatory processes has been observed in rodent models of stress-induced depression; for example, inflammatory cytokines such as interleukin (IL)-1β, IL-6, and IL-18 are increased, whereas anti-inflammatory cytokines such as IL-10, transforming growth factor (TGF)-β, and IL-4 are decreased (Liu et al., 2017; Zhang et al., 2021).

Microglia play an important role in the immunomodulation of the central nervous system (CNS). They exhibit mainly two types of polarity: M1-like (inflammatory) phenotype, which produces inflammatory mediators such as inducible nitric oxide synthase, IL-1β, and TNF-α, and M2-like (anti-inflammatory) phenotype, which increases the expression of anti-inflammatory cytokines such as IL-4, TGF-β, and IL-13 (Lan et al., 2017; Zhang et al., 2018). Activated microglia differentially affect the proliferation and differentiation of neural progenitor cells (NPCs) in vivo and in vitro. The inflammatory phenotype impairs NPC survival and proliferation, whereas the anti-inflammatory phenotype increases the generation of new neurons (Vay et al., 2018). Decreased neuronal cell survival in the dentate gyrus (DG) region of the adult hippocampus has been reported to be associated with depression in both rodents and humans. Additionally, the effects of long-term antidepressant use are believed to be exerted through the promotion of hippocampal neuroprotection (Castrén et al., 2007; Duman and Monteggia, 2006; Santarelli et al., 2003; Scorza et al., 2005). Anti-inflammatory agents, such as minocycline, have been shown to suppress the inflammatory phenotype of microglia, promote neurogenesis (Han et al., 2019; Takahashi et al., 2024b), and exhibit antidepressant effects in animal models of depression (Yang et al., 2020; Zheng et al., 2015). These findings suggest that modulating the switch in the microglial phenotype from neurotoxic to neurogenesis-promoting may contribute to antidepressant-like effects.

Olfactory bulbectomized (OBX) mice is an ideal animal model for studying depression. OBX mice exhibit a variety of abnormal behaviors, including depression-like behaviors and memory impairment (Nakagawasai et al., 2016; Takahashi et al., 2017, 2018a). Furthermore, physiological and neurochemical changes in the OBX model, such as decreased monoamine levels, cell proliferation, neuronal cell survival, and myelination and increased neuroinflammation in the brain, are similar to those observed in clinical depression (Takahashi et al., 2016, Takahashi et al., 2020a, Takahashi et al., 2020b, Takahashi et al., 2021Takahashi et al., 2016, 2018a, 2020a, 2020b, 2021, 2022).

Activation of adenosine monophosphate (AMP)-activated protein kinase (AMPK) by AMPK activators, such as metformin and resveratrol, has been shown to invoke antidepressant effects in patients with depression (Davinelli et al., 2017; Guo et al., 2014) and in animal models of depression (Ali et al., 2015; Fang et al., 2020; Takahashi et al., 2024a). Recently, we discovered that AMPK activators, such as 5-aminoimidazole-4-carboxamide-1-β-d-ribonucleotide (AICAR) and liver hydrolysate, exert preventive effects on animal models of depression by enhancing cell proliferation in hippocampal DG (Nakagawasai et al., 2020b, Nakagawasai et al., 2020c; Odaira et al., 2019). Other studies reported that AMPK activation modulates microglial polarity in the brain (Chen et al., 2025; Wang et al., 2018). Activation of AMPK has been shown to suppress neuroinflammation through the inhibition of nuclear factor kappa B activity (Liu et al., 2024) or by regulating autophagy (Takahashi et al., 2024a). Concurrently, its activation facilitates neurogenesis by enhancing the expression of brain-derived neurotrophic factor (BDNF) via the activation of cAMP response element binding protein (Mahmoud et al., 2023). However, it remains unknown whether microglia are involved in the antidepressant effects of AICAR in OBX mice.

Therefore, in the present study, we examined whether AICAR improves OBX-induced depression-like behaviors in mice and investigated its underlying molecular mechanisms from the perspective of neuroprotection.

## 2 Materials and methods

### 2.1 Animals

Male ddY mice (age, 6–7 weeks; weight, 26–28 g; Japan SLC, Shizuoka, Japan) were used in all the experiments (total, n = 125; behavioral test, n = 92; immunohistochemical study, n = 33). The mice were housed in cages under controlled environmental conditions (temperature, 23 ± 1 °C; humidity, 55 ± 5 %; and a 12/12 h light-dark cycle with lights on at 7:00), with five to six mice in each cage, and had unlimited access to food and water. They were subjected to behavioral testing between 9:00 and 17:00. The behavioral test was performed on each mouse only once. To prevent subjective bias, behavior was observed by a skilled observer blinded to the group for all behavioral tests, and statistical analysis of the data was conducted by a different experimenter. A stopwatch was used to measure the quantity of behavior in the behavioral tests, except in the sucrose preference test.

All animal experiments were approved by the Ethics Committee of Animal Experiments of Tohoku Medical and Pharmaceutical University (approval numbers: 19,022-cn and 20,051-cn). All animal experiments complied with the Animal Research: Reporting of In Vivo Experiments (ARRIVE) guidelines and were performed following the guidelines established by the Ethics Committee of Animal Experiments of Tohoku Medical and Pharmaceutical University and the United States National Institutes of Health Guide. Efforts were made to minimize suffering and the number of animals used.

### 2.2 Olfactory bulbectomy

OBX surgery was conducted as described previously (Nakagawasai et al., 2020a). All mice were euthanized at the end of the experiment, and we visually confirmed whether two-thirds of the olfactory bulbs (OBs) had been lesioned. Mice were excluded from the study if the lesion did not extend to more than two-thirds of the OBs or if it extended to the cortex. The sham operations followed the same surgical procedure without removal of the OBs.

### 2.3 Drugs

AICAR (100 mg/kg; Toronto Research Chemicals, Toronto, Canada) dissolved in saline was injected intraperitoneally at a volume of 0.1 mL/10 g body weight. This dose was based on a previous report (Odaira et al., 2019).

### 2.4 Tail suspension test (TST)

The TST was performed as previously described (Nakagawasai et al., 2020b, Nakagawasai et al., 2020c). Briefly, mice were taped from the tip of their tail and suspended at a height of 30 cm from the floor for 10 min. An observer blinded to the treatment allocation measured the immobility time for 10 min.

### 2.5 Forced swimming test (FST)

The FST was performed as previously described (Takahashi et al., 2018a, Takahashi et al., 2018b). Briefly, each mouse was individually placed on a vertical plastic cylinder (height, 25 cm; diameter, 20 cm) that contained water till a depth of 14 cm and was maintained at 25 °C for 5 min. The mouse was considered immobile when it floated passively in water and made the necessary movements to keep its head above the waterline. The immobility time was measured for 5 min by an observer blinded to the treatment allocation.

### 2.6 Sucrose preference test (SPT)

The SPT was performed as previously described (Nakagawasai et al., 2016). This experiment was conducted for 3 days, starting from 43 days post-OBX surgery. The training phase (day 1) lasted for at least 24 h and took place before the testing phase to allow the animals to adapt to the novel solution. At the beginning of the training phase, the animals were transferred to single housing with free access to food and two bottles of liquid, one containing 1 % sucrose solution and the other containing tap water. To prevent the possible effects of side preference on drinking, the position of the bottles was switched twice daily. The bottles were weighed every morning (9:00–11:00) to measure the amount of tap water and sucrose fluid consumed by the mice before and after the sucrose preference test period (days 2–3). Sucrose preference was calculated as the percentage of the consumed sucrose solution relative to the total volume (V) of the liquid consumed using the following formula: sucrose preference (%) = V (sucrose solution)/[V (sucrose solution) + V (water)] × 100. A decrease in sucrose preference, i.e., anhedonia, is a well-known indicator of a depression-like state in animals.

### 2.7 Sucrose splash test (SST)

The SST was performed as previously described (Takahashi et al., 2022). Briefly, each mouse was individually kept in a clear plexiglass box (height, 17 cm; width, 25 cm; length, 30 cm) for 30 min to allow it to adapt to the environmental conditions. Thereafter, a 10 % sucrose solution was sprayed onto the dorsal coat of the mouse. Because of its viscosity, the sucrose solution adheres to the mouse fur, and the animal initiates grooming behavior. After spraying the sucrose solution, the grooming time was manually recorded for 5 min as an index of self-care and motivational behavior. The apparatus was cleaned with a solution of 10 % ethanol between tests to remove any individual traces.

### 2.8 Immunohistochemistry

For immunohistochemical analysis, hippocampal samples were obtained from mice that had not undergone behavioral testing. After 21 days post-surgery, mice were injected with 5-bromo-2′-deoxyuridine (BrdU) (50 mg/kg i. p.; Nacalai Tesque, Inc., Kyoto, Japan) five times every 24 h to analyze neuronal cell survival in hippocampal DG. The animals were euthanized 24 h after the final AICAR injection. Brain samples were collected as previously described (Nakagawasai et al., 2022; Takahashi et al., 2019b). Briefly, mouse brains were sliced into 40-μm sections from −1.40 to −2.00 mm relative to bregma using a cryostat (Microm HM560; Microm International GmbH, Walldorf, Germany) and then frozen at −80 °C. Subsequently, the frozen sections were mounted on glass slides (Matsunami Glass, Osaka, Japan) and treated with hydrochloric acid (2 N) at 37 °C for 30 min, followed by neutralization with sodium borate buffer (0.15 M) at room temperature (23 ± 1 °C), twice for 10 min each, to facilitate better reactivity of anti-BrdU antibody. After washing the sections for 5 min thrice, they were incubated with phosphate-buffered saline (PBS) containing 1 % normal goat serum or 1 % normal donkey serum and 0.3 % Triton X-100 (PBSGT or PBSDT, respectively) at room temperature (23 ± 1 °C) for 2 h. They were then incubated overnight at 4 °C with primary monoclonal antibodies against rat anti-BrdU (1:100; Harlan SeraLab, Loughborough, UK), mouse anti-neuronal nuclear antigen (NeuN; 1:500; Millipore, Burlington, USA), rabbit anti-CD86 (1:200; Thermo Fisher Scientific, Waltham, USA), goat anti-CD206 (1:500; R&D Systems, Minneapolis, USA), and rabbit anti-ionized calcium binding adaptor molecule 1 (Iba1; 1:200, Wako Pure Chemical Industries Osaka, Japan). After incubating for 2 days, the sections were washed twice with 0.1 % PBS. When double labeling was performed using two primary antibodies from different host species (rabbit, mouse, rat, or goat), sections were washed and incubated overnight at 4 °C with goat anti-rat immunoglobulin G (IgG) Alexa Fluor 568 (1:200; Molecular Probes, Eugene, USA), goat anti-mouse IgG Alexa Fluor 488 (1:200; Molecular Probes), donkey anti-rabbit IgG Alexa Fluor 488 (1:200; Molecular Probes), or donkey anti-goat IgG Alexa Fluor 568 (1:200; Molecular Probes) with PBSGT or PBSDT. When double labeling was performed using two primary antibodies from the same host species (rabbit anti-Iba1 and rabbit anti-CD86 antibodies), each antigen was detected sequentially, and labeled goat anti-rabbit IgG Alexa Fluor 488 AffiniPure Fab fragment (1:80, Jackson ImmunoResearch Inc., West Grove, USA), instead of whole antibodies, was used for the first detection (Iba1). CD86 was then labeled with goat anti-rabbit IgG Alexa Fluor 488 (1:200; Molecular Probes). The immunohistochemical staining with two primary antibodies from the same host species was performed as previously described (Takahashi et al., 2019a; Yamagata et al., 2020). 4,6-diamidino-2-phenylindole (DAPI; 1:100; Wako Pure Chemical Industries, Ltd.) was used to stain and identify the nuclei. Sections were washed twice with 0.1 % PBS and coverslipped with a fluorescent mounting medium (Dako, Carpinteria, CA, USA). The labeled sections were analyzed under a confocal laser scanning microscope (A1Rsi; Nikon, Tokyo, Japan).

Three sections were collected from each mouse. Two images of the DG region of the hippocampus (i.e., left and right hemispheres, 640 × 640 μm) were obtained from each section. NeuN^+^/BrdU^+^ cells were counted based on the technique described by Ouchi et al. (2013). Positive cells in the region were counted using fluorescence microscopy. The number of positive cells in 2 images × 3 sections per mouse was added. The total amount of NeuN^+^/BrdU^+^ cells in the entire dorsal hippocampus was considered the total value. Moreover, we evaluated microglial polarization by counting Iba1^+^/DAPI^+^, CD86^+^/Iba1^+^, and CD206^+^/Iba1^+^ cells. Six images were analyzed per mouse and each group contained five to six mice.

### 2.9 Statistical analysis

The results are expressed as the mean ± standard error of the mean (SEM). Significant differences were determined using one-way analysis of variance (ANOVA), followed by Tukey-Kramer tests for comparisons among multiple groups using GraphPad Prism 7 (GraphPad Software Inc., La Jolla, CA, USA). Pearson's correlation coefficients (r = 0.2-weak, r = 0.5-moderate, r ≥ 0.8-strong) were calculated to determine the association between the NeuN^+^/BrdU^+^ cell count and Iba1^+^/CD86^+^ cell (%) or Iba1^+^/CD206^+^ cell (%). A p-value <0.05 was considered statistically significant.

## 3 Results

### 3.1 AICAR administration improved the OBX-induced depression-like behaviors

**Fig. 1:**
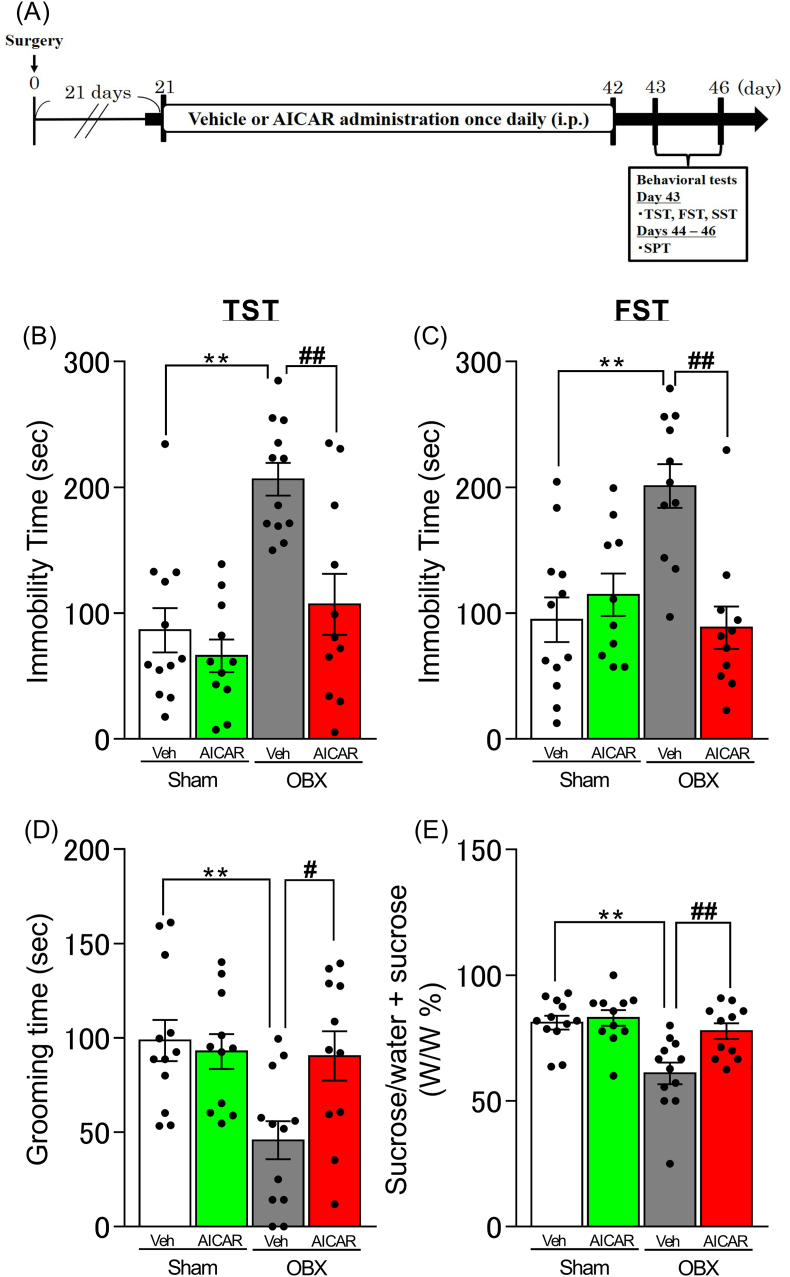
Effect of AICAR on depression-like behaviors in OBX mice. A: Time course of experimental protocol. Duration of immobility in TST (B) and FST (C) and grooming time in SST (D). E: Rate of sucrose intake in the SPT. Two-way ANOVA: group × treatment: F (1, 42) = 5.187, p = 0.0279, group: F (1, 42) = 11.92, p = 0.0013, treatment: F (1, 42) = 21.45, p < 0.0001, (B); group × treatment: F (1, 40) = 14.68, p = 0.0004, group: F (1, 40) = 7.205, p = 0.0105, treatment: F (1, 40) = 5.363, p = 0.0258, (C); group × treatment: F (1, 42) = 5.339, p = 0.0258, group: F (1, 42) = 3.161, p = 0.0826, treatment: F (1, 42) = 6.43, p = 0.0150, (D); group × treatment: F (1, 42) = 4.797, p = 0.0341, group: F (1, 42) = 7.448, p = 0.0092, treatment: F (1, 42) = 14.02, p = 0.0005, (E). Bars represent means ± SEM. ∗∗p < 0.01 vs. sham + vehicle group. #p < 0.05, ##p < 0.01, vs. the OBX + vehicle group (n = 10–12 per group).

Compared with the sham control group, OBX mice exhibited a prolonged duration of immobility in the TST (p < 0.0001) and FST (p = 0.0004) and decreased grooming time (p = 0.0059) and sucrose intake rate (p = 0.0006) in the SST and SPT, respectively. However, these changes were improved by chronic administration of AICAR (TST: p = 0.0012, FST: p = 0.0002, SST: p = 0.0297, SPT: p = 0.0063) (Fig. 1).

### 3.2 AICAR prevented the reduction of neuronal cell survival in the hippocampus of DG in OBX mice

**Fig. 2:**
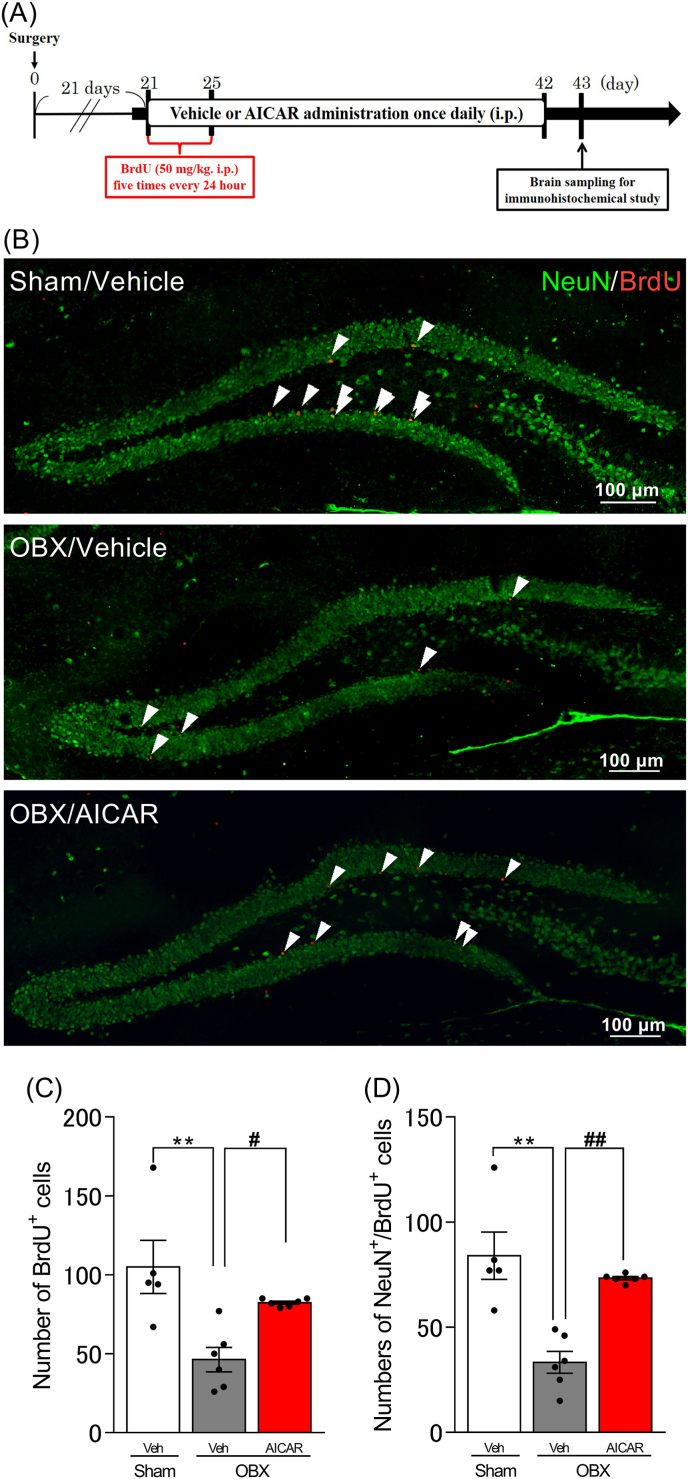
Effect of AICAR on neuronal cell survival in the hippocampal DG of OBX mice. A: Time course of experimental protocol. B: Confocal images of brain slices stained with NeuN (green) and BrdU (red). The arrowheads indicate double-positive cells in the DG. C and D: Quantitative analysis of the number of BrdU^+^ (C) and NeuN^+^/BrdU^+^ (D) cells in the hippocampal DG. One-way ANOVA: F (2, 14) = 9.098, p = 0.0029, (C); F (2, 14) = 16.82, p = 0.0002, (D). Bars represent means ± SEM. ∗∗p < 0.01 vs. sham + vehicle group. ##p < 0.01 vs. OBX + vehicle group (n = 5–6 per group). (For interpretation of the references to colour in this figure legend, the reader is referred to the Web version of this article.)

OBX mice were administered BrdU to detect the changes in hippocampal neuronal cell survival. Anti-NeuN antibody was used to identify mature neurons in the DG area. The incorporation of BrdU into cells indicated that they were actively dividing at the time of BrdU injection. OBX mice showed a significantly lower number of BrdU^+^ (p = 0.0002) and NeuN^+^/BrdU^+^ (p < 0.0001) cells in than in sham mice, while these changes were improved by chronic administration of AICAR (BrdU^+^: p = 0.0014, NeuN^+^/BrdU^+^: p < 0.0001) (Fig. 2).

### 3.3 AICAR prevented the polarization to M1-type microglia in the hippocampus of OBX mice

**Fig. 3:**
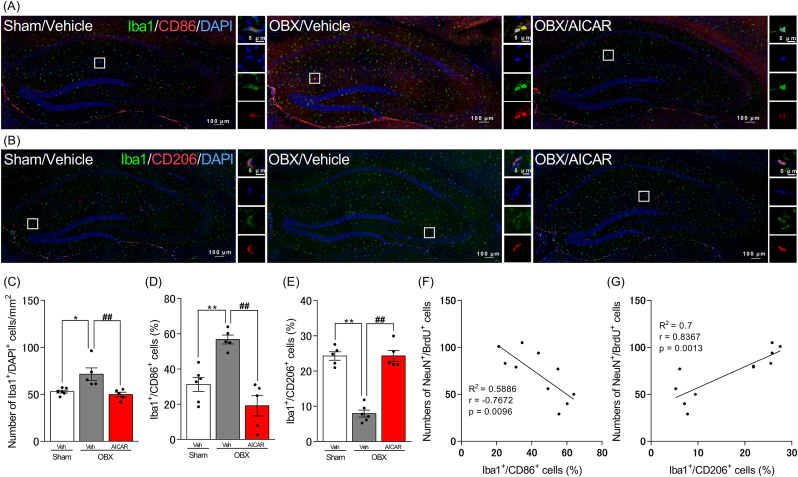
Effect of AICAR on hippocampal polarization of microglia in OBX mice. A and B: Confocal images of brain slices stained with DAPI (blue), Iba1 (green), CD86 (red) (A), or CD260 (red) (B). The boxed area is shown at higher magnification. C–E: Quantitative analysis of the number of Iba1^+^/DAPI^+^ cells (C) and the percentage of Iba1^+^/CD86^+^ (D) and Iba1^+^/CD206^+^ (E) cells in the hippocampus. F and G: Relationship between number of NeuN^+^/BrdU^+^ cells in the hippocampus of DG and the percentage of Iba1^+^/CD86^+^ cells (F) and Iba1^+^/CD206^+^ cells (G). One-way ANOVA: F (2, 13) = 8.617, p = 0.0041, (C); F (2, 13) = 19.3, p = 0.0001, (D); F (2, 13) = 61.39, p < 0.0001, (E). Bars represent means ± SEM. ∗p < 0.05, ∗∗p < 0.01 vs. sham + vehicle group. ##p < 0.01 vs. OBX + vehicle group (n = 5–6 per group). (For interpretation of the references to colour in this figure legend, the reader is referred to the Web version of this article.)

Anti-CD86 and anti-CD206 antibodies were used to identify the M1-and M2-like phenotypes, respectively. OBX mice showed higher Iba1^+^/DAPI^+^ (p = 0.0123) and CD86^+^/Iba1^+^ (p = 0.0023) cells and lower CD206^+^/Iba1^+^ (p < 0.0001) cells in the hippocampus compared with the sham group, whereas these changes were reversed by chronic AICAR treatment (Iba1^+^/DAPI^+^: p = 0.0057, CD86^+^/Iba1^+^: p = 0.0001, CD206^+^/Iba1^+^: p < 0.0001) (Fig. 3A–E). In addition, linear regression showed that the number of NeuN^+^/BrdU^+^ cells in the hippocampus of DG was significantly negatively correlated with the percentage of Iba1^+^/CD86^+^ cells (Fig. 3F) and significantly positively correlated with the percentage of Iba1^+^/CD206^+^ cells (Fig. 3G).

## 4 Discussion

Several studies have reported that the activation of AMPK in the CNS through exercise or AMPK activators, including AICAR, resveratrol, or metformin, alleviates depression symptoms in patients (Guo et al., 2014) and animal models of depression (Odaira et al., 2019; Shen et al., 2020). In our previous study, we demonstrated that AICAR has a preventive effect on depression-like behaviors in OBX mice (Odaira et al., 2019). However, the mechanism by which AMPK activation improves OBX-induced depression-like behavior remains unknown. In the present study, we found that OBX mice exhibited depression-like behaviors in the TST, FST, SST and SPT, respectively, which are consistent with the findings of previous studies (Nakagawasai et al., 2016, 2020c; Takahashi et al., 2022). The age of the mice utilized in this study corresponds to the adolescent stage, a period during which psychiatric disorders frequently manifest clinically (Takahashi et al., 2019b; Weersing et al., 2016). In addition, OBX-induced depression-like behaviors were improved by chronic administration of AICAR. In our previous study, we found that administration of AICAR at the same dose used in this study activated AMPK in the hippocampus of OBX mice (Odaira et al., 2019); thus, we assumed that AMPK was activated in the present study as well. Furthermore, the dosage of AICAR employed in this study has been documented to exert an antidepressant effect in a model of depression induced by a high-fat diet and corticosterone treatment (Liu et al., 2014). Therefore, we suggest that chronic AMPK activation improves OBX-induced depression-like behavior.

Reduced neuronal cell survival in the hippocampus contribute to the development of depression in both rodents and humans (Castrén et al., 2007; Duman and Monteggia, 2006; Scorza et al., 2005), and improving this cell survival is associated with antidepressant effects (Santarelli et al., 2003; Tartt et al., 2022). Our previous study demonstrated that AICAR administration prevented the OBX-induced decrease in cell proliferation in the DG region of the hippocampus, and inhibiting the effect of AICAR on cell proliferation resulted in the abolishment of the antidepressant effects of AICAR (Odaira et al., 2019). Thus, we hypothesized that the antidepressant effects of AICAR may also be associated with neuroprotection in the hippocampal DG of OBX mice. Therefore, animals were injected with BrdU on day 21–25 after surgery to determine the rate of hippocampal neuronal cell survival. We found the reduction of neuronal cell survival in the hippocampal DG in OBX mice, which is consistent with the results of previous studies (Moriguchi et al., 2020; Xu et al., 2018), while these changes were improved by chronic administration of AICAR in the present study. In the present study, we measured the number of BrdU^+^ cells in the hippocampal DG after 3 weeks of BrdU administration to assess cell survival and found decreased cell survival in the hippocampal DG of OBX mice. In contrast, elevated proportion of BrdU^+^ cells in the hippocampal DG 24 h after BrdU administration indicates cell proliferation. In our previous study, we observed decreased cell proliferation in the hippocampal DG of OBX mice 6 weeks after surgery (Takahashi et al., 2018a). OBX mice at 6 weeks after surgery showed increased expression levels of inflammatory cytokines (TNF-α and IL-6), indicating cell injury, and decreased expression of BDNF, which promotes cell proliferation, in the hippocampus (Takahashi et al., 2018a). Thus, these findings suggest that OBX suppresses cell proliferation and enhances cell degeneration in the hippocampus. Therefore, these results suggested that the antidepressant effects of AICAR in OBX mice may be associated with enhanced hippocampal neuroprotection.

Microglial polarity can be divided into classical M1-like (inflammatory) and alternative M2-like (anti-inflammatory) phenotypes. The M1-like microglia produce inflammatory mediators (Zhang et al., 2018) and suppresses hippocampal neurogenesis (Jin et al., 2014), while the M2-like microglia enhance the expression of anti-inflammatory cytokines and promote neuroprotection (Lan et al., 2017). The activation of AMPK influences the transition between M1 and M2 phenotypes in microglia within the brain, thereby eliciting anti-inflammatory effects (Chen et al., 2025; Wang et al., 2018). In a previous study, we found that the expression of M1-type microglial markers such as TNF-α and IL-6 was increased while that of the M2-type microglial marker BDNF was decreased in the hippocampus of OBX mice after 6 weeks of surgery, along with an increase in the number of Iba1-positive cells (Takahashi et al., 2018a). These results suggest that OBX mice have microgliosis and microglial polarity imbalances in the hippocampus. Increased phosphorylation of AMPK in microglia has been reported to suppress the production of inflammatory cytokines (TNF-α and IL-6) and promote the production of BDNF (M2-type microglia marker) (Bolós et al., 2017; Lai et al., 2018; Takahashi et al., 2024a). Since TNF-α is known to activate resting microglia to M1-type (Bolós et al., 2017), AMPK activation is considered to reduce the production of TNF-α, thereby suppressing polarization to M1-type. We subsequently found that AICAR-induced phosphorylation of AMPK is colocalized with Iba1 (microglial marker) or NeuN (mature neuron marker) in the hippocampus of OBX mice (Odaira et al., 2019). Thus, we hypothesized in the present study that the administration of AICAR to OBX mice will normalize the microglial polarization, resulting in the promotion of neuroprotection in the hippocampal DG. In the present study, we found that OBX mice showed higher Iba1^+^/DAPI^+^ and CD86^+^/Iba1^+^ cells and lower CD206^+^/Iba1^+^ cells in the hippocampus, whereas these changes were prevented by chronic AICAR treatment. Moreover, linear regression showed that the number of NeuN^+^/BrdU^+^ cells in the hippocampus of DG was significantly negatively correlated with the percentage of Iba1^+^/CD86^+^ cells and significantly positively correlated with the percentage of Iba1^+^/CD206^+^ cells, suggesting a close relationship between microglial polarization and neurogenesis in the hippocampus of DG. These findings suggest that AMPK activation exerts anti-inflammatory effects by regulating the polarization of microglial M1/M2 phenotypes.

Despite these novel findings, this study has some limitations. The prevalence and severity of depression are higher in women than in men. However, female rodents have an estrus cycle, and changes in hormonal balance are known to affect behavioral, pharmacological, and biochemical outcomes (Carrier et al., 2015; Giacometti et al., 2022). In addition, the antidepressant effect of fluoxetine, a selective serotonin reuptake inhibitor, has been found to be attenuated in female mice during estrus (Yohn et al., 2020). Hence, in the present study, to avoid the influence of the estrus cycle on the results, we used only male mice to evaluate the efficacy of AICAR, an AMPK activator. However, studying the efficacy of AICAR in female OBX mice is also essential, and we aim to conduct further research on this topic based on the results of this study.

## 5 Conclusions

In conclusion, this study revealed that AICAR exerts antidepressant effects in OBX mice by preventing OBX-induced disturbance of microglial polarization in the hippocampus. Our findings suggest that AMPK activators and behaviors that can activate AMPK such as regular exercise have therapeutic potential for neuropsychiatric disorders such as depression. However, it remains unknown whether neuroprotection of the hippocampus is directly involved in the antidepressant effects of AICAR. Therefore, in future studies, we plan to focus on hippocampal neuroprotection caused by AICAR.

## CRediT authorship contribution statement

**Takayo Odaira-Satoh:** Writing – original draft, Visualization, Investigation, Formal analysis. **Osamu Nakagawasai:** Validation, Methodology, Conceptualization, Project administration, Writing – review & editing, Funding acquisition. **Kohei Takahashi:** Writing – original draft, Visualization, Investigation, Funding acquisition. **Masae Shimada:** Investigation. **Wataru Nemoto:** Investigation. **Koichi Tan-No:** Supervision, Writing – review & editing, Validation, Project administration, Methodology, Funding acquisition, Conceptualization.

## 6 Funding

This study was supported in part by Grants-in-Aid for Scientific Research [grant numbers 22K06866 and 24K18367].

## Declaration of competing interest

The authors declare no competing interest.

## Data Availability

Data will be made available on request.
